# The association of work stress and night work with nutrient intake – a prospective cohort study

**DOI:** 10.5271/sjweh.3899

**Published:** 2020-09-01

**Authors:** Katri Hemiö, Jaana Lindström, Markku Peltonen, Mikko Härmä, Katriina Viitasalo, Sampsa Puttonen

**Affiliations:** 1 Department of Public Health Solutions, Finnish Institute for Health and Welfare, Helsinki, Finland; 2 Research and Service Centre of Occupational Health, Finnish Institute of Occupational Health, Helsinki, Finland; 3 Finnair Health Services, Vantaa, Finland

**Keywords:** airline, diet quality, Epworth Sleepiness Scale, fat intake, saturated fat intake, shift work, shift worker

## Abstract

**Objectives:**

In a prospective study among workers in an airline company, we explored whether change in work stress symptoms or night shifts was associated with nutrient intake.

**Methods:**

Participants in a workplace type 2 diabetes (T2D) prevention study completed a questionnaire on lifestyle, work stress symptoms, work schedule, and food intake at baseline and after 2.4-years follow-up (211 men and 155 women, 93% with increased risk for T2D). Multiple linear regression models with covariates were used to explore the associations between change in work stress symptoms or night shifts and change in nutrient intake during the follow-up.

**Results:**

Among men, an increase in stress and a decrease in perceived workability was associated with a higher proportion of energy (E%) from fat [β 0.6, 95% confidence interval (CI) 0.07–1.11, β 1.3, 95% CI 0.57–2.05] and saturated fat (β 0.3, 95% CI 0.02–0.58, β 0.5, 95% CI 0.14–0.90), respectively. Furthermore, a decrease in workability was associated with lower vitamin C intake (β -9.2, 95% CI -16.56– -1.84) and an increase in sleepiness with higher E% from saturated fat (β 0.7, 95% CI 0.00–0.15). Among women, an increase in work-related fatigue was associated with higher alcohol intake (β 7.5, 95% CI 1.25–13.74) and an increase of night shifts was associated with higher E% from fat (β 0.24, 95% CI 0.00–0.47) and saturated fat (β 0.17, 95% CI 0.04–0.29).

**Conclusions:**

Work stress symptoms were associated with a reduction in diet quality especially among men. The possible impact of work stress symptoms on workers’ dietary habits should be acknowledged and the assessment of dietary habits should consequently be incorporated into occupational health examinations.

Over the last decades, weekly working hours have decreased, but at the same time the experience of high work demands has mostly increased in Europe (1). In addition, atypical working times have become more common. The prevalence rate of shift work increased from 17% to 21% between 2005 and 2015 (2). Of all employees in Europe, 25% are estimated to experience work-related stress (3). Due to changes in work environment and conditions, workers experience more work-related strains, which can be detrimental for health in a long term.

Work stress is mainly caused by psychosocial factors such as high work demands, low job control, and irregular working hours. Long-term exposure to work stress increased the risk for coronary heart diseases and weight gain (4–6). Among shift workers, insomnia, sleep deprivation and daytime sleepiness are more common than among dayworkers (7, 8). The risk for cardiovascular diseases and type 2 diabetes is increased in shift workers (9, 10) but also among short-duration or poor sleepers (11–13).

One study showed that a third of the associations between work stressors and cardiovascular diseases may be explained by impaired lifestyle habits such as poor dietary habits (4). Two studies showed longitudinal association of stress with dietary habits: high work-related stress predicted lower vegetable consumption (4) and stress due to situations in life predicted higher fast food consumption (14). Longitudinal studies of work stress and specific nutrient intake are lacking. In cross-sectional studies, associations of work stress with higher fat intake and lower carbohydrate intake were seen in both sexes (15), but two studies found an association of higher fat intake only among men (16, 17). These studies suggest that sex differences may exist in associations between work stress and diet. Workers’ perceived stress at work and disturbed sleep are closely related (18, 19). Better sleep patterns are associated with better diet quality (20, 21) and short sleep duration with poor dietary habits, higher energy intake and risk for obesity (22–24). Also shift work may affect workers’ dietary habits. Based on cross-sectional studies, night shift is associated with lower fruit and – in some – micronutrients intake and higher energy intake (25, 26) and unhealthy eating habits strengthened with more years in night shift work (27). Recent review article concludes that shift work impairs dietary quality by increasing saturated fat and sugar consumption based on cross-sectional studies (28), but the effect of change in shift schedule on diet is not known.

Therefore, we aimed to determine if changes in work stress – including subjective symptoms of work stress, sleepiness or night work – were associated prospectively with nutrient intake. As men and women may differ in their reactions to stress (29), the analyses were stratified by sex.

## Methods

### Study design and participants

A Finnish airline company had a policy to invite one fifth of employees to a health check-up each year. In 2006, the company initiated a type 2 diabetes (T2D) prevention program (30). In total, 4169 employees (60% of the workforce) were invited without inclusion criteria, of whom 2312 employees (67% working in shifts) participated in health check-ups organized by the occupational healthcare unit. Two and half years later, the baseline health check-up participants were invited to a follow-up health check-up, in which 1347 workers participated (31). Workers with T2D identified at the baseline check-up were treated according to standard procedures and were excluded from the study. The Ethics Committee of the Hospital District of Helsinki and Uusimaa approved the study, and all participants gave their written informed consent.

**Figure 1:**
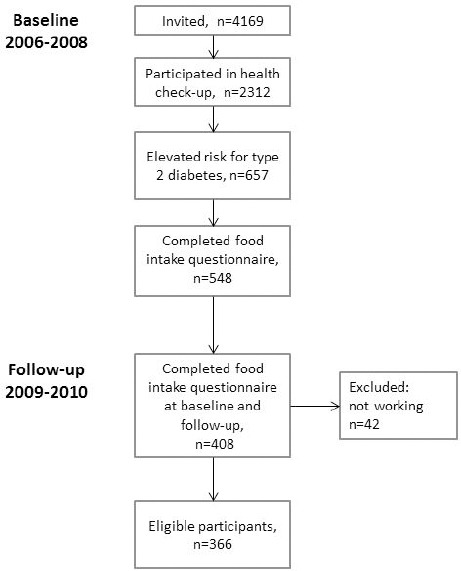
Flowchart of participants

As part of the health check-ups, participants were asked to complete comprehensive questionnaire containing questions about lifestyle, work, sleep, and diabetes risk factors (FINDRISC) (32). In addition, participants with elevated risk for T2D (N=657), based on FINDRISC score and plasma glucose measurements, were asked to complete a food intake questionnaire (33) and offered two face-to-face lifestyle counselling sessions aiming at lowering their risk for chronic diseases. Sessions were focussed on dietary and exercise habits, weight management and influence of work schedule on lifestyle. Altogether 408 participants completed the food intake questionnaire both at baseline and follow-up, on average 2.4 years apart. Of the participants, 42 were excluded because they had stopped working for the company or retired. The final analytical sample thus consisted of 366 participants (figure 1).

### Nutrient intake

In a food intake questionnaire, participants were asked to estimate their usual consumption of 16 different food groups (33). As it has been developed as a tool for chronic disease, especially for T2D prevention in primary healthcare, the food intake questionnaire covers the food groups that are mostly associated with chronic diseases. The questionnaire has been validated against 7-day food diaries, and algorithms have been created to estimate nutrients relevant to chronic disease prevention including total fat (proportion of energy, E%), saturated fat (E%), sucrose (E%), fiber (g), vitamin C (mg), iron (mg) and vitamin D (μg) (33). Alcohol consumption was assessed with a question: “How much alcohol do you drink weekly?” Portions of beer, wine and spirits were converted into grams per alcohol in a week.

### Classification and covariate variables

Education was categorized into low (comprehensive school), intermediate (secondary education) or high (polytechnic or university). Body mass index was calculated by dividing weight (kg) by height (m) squared. Occupations were categorized based on characteristics of the work into maintenance and loading, customer service, in-flight, office, and management work. Marital status was categorized into living alone or cohabitating.

Stress was assessed with the question: “Stress means a situation in which a person feels tense, restless, nervous or anxious or is unable to sleep at night because his/her mind is troubled all the time. Do you feel this kind of stress?” Answering alternatives were: 0=not at all, 1=some, 2=rather much, 3=very much (34).

Work-related fatigue was assessed with the question: “I feel exhausted from work”. The question was rated on a 4-point scale: 0=not at all, 1=somewhat, 2=rather a lot, 3=very much. Perceived workability in the future was assessed with the question: “Do you believe considering your health you could work in your current job after two years?” The question was rated on a 3-point scale: 0=rather sure, 1=not sure, 2=unlikely (35).

Sleepiness was assessed with the Epworth Sleepiness Scale (ESS) (36) containing eight questions that were rated on a 4-point Likert-type scale: 0=would never doze, 1=slight, 2=moderate, 3=high chance of dozing.

Day work was defined as work performed between 06:00–18:00 hours and shift work was defined as work other than day work. Night shift was defined as work performed at ≥3 hours between 23:00–06:00 hours.

Low diabetes risk was defined as FINDRISC (32) score <10 and fasting plasma glucose <6.1 mmol/l and elevated diabetes risk when FINDRISC score was ≥10 or fasting plasma glucose was 6.1–6.9 mmol/l or the 2-h glucose in the oral glucose tolerance test was 7.8–11.0 mmol/l. The FINDRISC score consists of 8 items including questions of age, body mass index, waist circumference, physical activity, consumption of fruit and vegetables, hypertension medication, history of high blood glucose, and family history of diabetes and gives an estimate of the subject’s probability to get diabetes in ten years.

### Statistical methods

We calculated the change from baseline to follow-up in nutrient intake and stress-related variables by subtracting baseline value from follow-up value. Using paired student’s t-test, we compared separately the nutrient intake change from baseline to follow-up of lifestyle intervention participants, non-participants, men, and women. In addition, using linear regression, we analyzed the difference in nutrient intake change during follow-up between lifestyle intervention participants and non-participants. We analyzed separately the association of work-related variables change (independent variables: stress, fatigue, workability, ESS, and night work) with the change of nutrient intake (dependent variable) using multiple linear regression analysis stratified by sex. The analyses were based on the assumption that every unit change in the independent variable results in the same increase/decrease in the dependent nutrient intake variables. This was verified by graphical means eg, scatter plots. The analyses were adjusted for baseline nutrient intake and participation in lifestyle intervention. In addition, we further adjusted analyses for age (continuous), education (3 groups), work schedule (day versus shift work), full versus part time work, occupation (5 groups) and marital status. The work schedule was not included in the analyses of night shift work and nutrients. The covariates were chosen because of their direct and indirect relationships with both stress-related variables and nutrition. The significance level was set at α=0.05. All analyses were calculated using STATA software, version 15.1 (Stata Corp, College Station, TX, USA).

## Results

Participants’ baseline characteristics are shown in table 1 separately for men and women. Of the participants, 58% were men and shift work was common among both men (53%) and women (49%). A majority of the participants, 84% of men and 93% of women, felt that they were able to work in their current work after two years. Two-thirds of the participants took part in lifestyle interventions. Changes in nutrient intake during the follow-up among participants and non-participants as well as among men and women are shown in the supplementary material (www.sjweh.fi/show_abstract.php?abstract_id=3899) table S1. Participants’ and non-participants’ nutrient intake changes did not differ. During the follow-up 6% of men and 4% of women changed their work schedule between shift and day work. Among men, the number of night shifts increased by 9% and among women by 19% during the follow-up. Perceived stress was reported in 53% and 57% of men and women, respectively. During the follow-up, 27% of men and 31% of women felt that their stress had increased.

**Table 1:** Study participants’ characteristics at baseline. [SD=standard deviation.]

	Men (N=211)		Women (N=155)	
	
	%	Mean (SD)	%	Mean (SD)
Sex	58		42	
Age		48.9 (6.8)		47.3 (7.1)
Married or cohabitating	81		67	
Education ^a^				
Low	14		12	
Intermediate	77		73	
High	9		15	
Body mass index		29.1 (3.8)		28.5 (5.2)
No lifestyle invention	27		31	
Work characteristics				
Maintenance and loading	55		10	
Customer service	1		20	
In-flight work	6		19	
Office work	23		45	
Management	15		5	
Full time work	98		93	
Shift work	53		49	
Regular	49		15	
Including night shifts	44		45	
Including early morning (<06:00 hr)	40		67	
Night shifts / month (shift workers)		5 (2.5)		3 (1.5)
Feeling stress	53		57	
Fatigue due to work	13		14	
Future workability (unsure or unlikely)	16		7	
Epworth sleepiness scale ^b^		5.7 (3.2)		6.3 (3.8)

a Low= comprehensive school, intermediate=high school, vocational school or college, high=polytechnic

b Range for men 0–17 and for women 0–18.

Among men, increased stress was associated with increased fat [β=0.59, 95% confidence interval (CI) 0.07–1.11, P=0.03] and saturated fat intake (β=0.31, 95% CI 0.02–0.58, P=0.02) (table 2). Reduced workability was associated with increased fat (β=1.31, 95% CI 0.57–2.05, P<0.001) and saturated fat intake (β=0.52, 95% CI 0.14 –0.90, P=0.005) and a decrease in vitamin C intake (β=-9.20, 95% CI -16.56– -1.84, P=0.03). Moreover, sleepiness associated with an increase in saturated fat (β=0.07, CI 0.00–0.15, P=0.03) All the above results were controlled for confounders (age, education, work schedule, full versus part time work, occupation, and marital status).

**Table 2:** Association between changes in work stress symptoms or number of night shifts and in nutrient intake during 2.4 years follow-up among **men** (N=211) [ß=regression coefficient; CI=confidence interval].

Change in:		Feeling stress ^a^		Fatigue ^a^		Workability ^a^		Epworth Sleepiness Scale ^a^		Number of night shifts ^a^	
				
		ß ^b^	95% CI	ß ^b^	95% CI	ß ^b^	95% CI	ß ^b^	95% CI	ß ^b^	95% CI
Sucrose (E%)	Model 1 ^c^	0.02	-0.32–0.35	0.17	-0.15–0.48	-0.17	-0.65–0.31	0.04	-0.06–0.13	0.04	-0.12–0.19
	Model 2 ^d^	-0.07	-0.41–0.27	0.10	-0.22–0.42	-0.19	-0.68–0.30	0.04	-0.06–0.13	0.03	-0.12–0.19
Fat (E%)	Model 1 ^c^	0.58	0.07–1.08 ^e^	0.08	-0.40–0.57	1.22	0.51–1.93 ^f^	0.10	-0.05–0.24	-0.19	-0.47–0.08
	Model 2 ^d^	0.59	0.07–1.11 ^e^	0.11	-0.39–0.61	1.31	0.57–2.05 ^g^	0.09	-0.05–0.24	-0.18	-0.46–0.10
Saturated fat (E%)	Model 1 ^c^	0.32	0.05–0.58 ^e^	0.08	-0.18–0.34	0.52	0.16–0.89 ^f^	0.08	0.01–0.15 ^e^	-0.03	-0.17–0.11
	Model 2 ^d^	0.31	0.02–0.58 ^e^	0.05	-0.22–0.32	0.52	0.14–0.90 ^f^	0.07	0.00–0.15 ^e^	-0.04	-0.18–0.11
Alcohol (g)	Model 1 ^c^	8.94	-4.93–22.81	4.44	-8.77–17.64	-3.01	-22.97–16.96	-0.10	-3.99–3.79	-0.87	-7.64–5.89
	Model 2 ^d^	9.60	-4.90–24.09	3.94	-10.01–17.89	-5.23	-26.50–16.04	-0.21	-4.28–3.86	-1.52	-8.53–5.49
Fibre (g)	Model 1 ^c^	-0.18	-0.88–0.53	-0.40	-1.13–0.34	-0.17	-1.19–0.86	-0.15	-0.37–0.07	0.16	-0.21–0.52
	Model 2 ^d^	-0.11	-0.83–0.62	-0.27	-1.04–0.50	0.07	-1.00–1.15	-0.13	-0.36–0.10	0.26	-0.16–0.69
Vitamin D (µg)	Model 1 ^c^	-0.17	-0.48–0.14	0.21	-0.08–0.51	-0.10	-0.54–0.34	-0.09	-0.17– -0.00 ^e^	-0.14	-0.28–0.01
	Model 2 ^d^	-0.18	-0.50– 0.14	0.25	-0.06–0.57	-0.09	-0.56–0.38	-0.09	-0.18– -0.00	-0.15	-0.39–0.10
Vitamin C (mg)	Model 1 ^c^	-5.03	-9.67– -0.39 ^e^	-3.01	-7.17–1.15	-8.01	-14.99– -1.02 ^e^	-1.32	-2.62– -0.01 ^e^	-1.33	-3.63–0.98
	Model 2 ^d^	-4.63	-9.46–0.20	-2.88	-7.20–1.44	-9.20	-16.56–- 1.84 ^e^	-1.21	-2.56–0.15	-2.00	-4.38–0.45
Fe (mg)	Model 1 ^c^	0.01	-0.23–0.23	-0.02	-0.29–0.24	0.29	-0.06–0.65	-0.04	-0.12–0.04	0.02	-0.11–0.15
	Model 2 ^d^	0.05	-0.21–0.30	0.00	-0.28–0.28	0.31	-0.06–0.68	-0.03	-0.12–0.04	0.07	-0.08–0.21

a Change from baseline was calculated subtracting follow-up value from baseline value. Range = change in stress level: -3–2, fatigue: -3–2, workability: -2–2, Epworth Sleepiness Scale: -13–10, and number of night shifts: -10–10.

b Positive slope indicate increase and negative slope decrease in nutrient intake when work stress symptom has increased.

c Adjusted for participation in lifestyle interventions and baseline nutrient intake.

d Further adjusted for age, education, work schedule, occupation, full or part time work at baseline or follow-up, and marital status except work schedule was not included in analysis of night shifts.

e P<0.05.

f P<0.01.

g P<0.001.

Among women, an increase in night shifts was associated with an increase in intake of fat (β=0.24, 95% CI 0.00–0.47, P=0.046) and saturated fat (β=0.17, 95% CI 0.04–0.29, P=0.02), and an increase in fatigue was associated with an increase in alcohol consumption (β=7.50, 95% CI 1.25–13.74, P=0.02) (table 3) when the results were controlled for confounders.

**Table 3:** Association between changes in work stress symptoms or number of night shifts and in nutrient intake during 2.4 years follow-up among **women** (N=155) [ß= regression coefficient; CI=confidence interval].

Change in:		Feeling stress ^a^		Fatigue ^a^		Workability ^a^		Epworth Sleepiness Scale ^a^		Number of night shifts ^b^	
				
		ß ^b^	95% CI	ß ^b^	95% CI	ß ^b^	95% CI	ß ^b^	95% CI	ß ^b^	95% CI
Sucrose (E%)	Model 1 ^c^	0.32	0.03–0.61 ^e^	-0.03	-0.32–0.27	-0.07	-0.67–0.53	0.01	-0.08–0.10	0.06	-0.09–0.21
	Model 2 ^d^	0.29	-0.01–0.60	-0.07	-0.38–0.23	-0.19	-0.80–0.43	0.01	-0.08–0.11	0.07	-0.08–0.22
Fat (E%)	Model 1 ^c^	-0.06	-0.53–0.41	-0.01	-0.48–0.46	0.22	-0.73–1.18	0.02	-0.12–0.17	0.21	-0.02–0.45
	Model 2 ^d^	-0.15	-0.63–0.34	-0.08	-0.56–0.40	0.15	-0.81–1.12	0.02	-0.12–0.16	0.24	0.00–0.47 ^e^
Saturated fat (E%)	Model 1 ^c^	0.01	-0.24–0.26	0.06	-0.20–0.31	0.05	-0.47–0.57	0.01	-0.07–0.09	0.15	0.03–0.28 ^e^
	Model 2 ^d^	-0.03	-0.29–0.23	0.03	-0.23–0.29	-0.00	-0.53–0.52	0.01	-0.07–0.09	0.17	0.04–0.29 ^e^
Alcohol (g)	Model 1 ^c^	-0.62	-6.99–5.75	7.66	1.49–13.84 ^e^	3.14	-9.31–15.60	1.11	-0.86–3.09	-1.03	-4.30–2.24
	Model 2 ^d^	-0.73	-7.32–5.85	7.50	1.25–13.74 ^e^	3.92	-8.61–16.45	0.86	-1.12–2.84	-1.50	-4.79–1.81
Fibre (g)	Model 1 ^c^	0.34	-0.17–0.84	-0.22	-0.73–0.30	0.86	-0.17–1.88	0.06	-0.09–0.22	-0.11	-0.37–0.14
	Model 2 ^d^	0.28	-0.24–0.81	-0.25	-0.77–0.28	0.75	-0.28–1.79	0.08	-0.09–0.23	-0.14	-0.39–0.12
Vitamin D (µg)	Model 1 ^c^	-0.22	-0.49–0.05	0.04	-0.24–0.31	0.41	-0.14–0.96	0.01	-0.08–0.09	-0.11	-0.25–0.02
	Model 2 ^d^	-0.20	-0.48–0.08	0.04	-0.24–0.32	0.46	-0.10–1.02	0.01	-0.08–0.09	-0.12	-0.26–0.02
Vitamin C (mg)	Model 1 ^c^	-1.34	-4.84–2.16	-2.76	-6.36–0.84	-1.32	-8.39–5.75	-0.48	-1.56–0.61	-0.77	-2.55–1.01
	Model 2 ^d^	-1.98	-5.59- 1.62	-3.21	-6.83–0.41	-1.75	-8.90–5.40	-0.46	-1.54–0.62	-0.83	-2.61 – 0.95
Fe (mg)	Model 1 ^c^	0.14	-0.08–0.35	-0.03	-0.25–0.19	0.25	-0.18–0.68	0.05	-0.02–0.11	-0.02	-0.12–0.09
	Model 2 ^d^	0.10	-0.12–0.33	-0.05	-0.27–0.17	0.21	-0.23–0.65	0.04	-0.02–0.11	-0.03	-0.14–0.08

a Positive slope indicate increase and negative slope decrease in nutrient intake when work stress symptom has increased.

b Change from baseline was calculated subtracting follow-up value from baseline value. Range = change in stress level: -3–2, fatigue: -3–2, workability: -2–2, Epworth Sleepiness Scale: -13–10, and number of night shifts: -10–10.

c Adjusted for participation in lifestyle interventions and baseline nutrient intake.

d Further adjusted for age, education, work schedule, occupation, full or part time work at baseline or follow-up, and marital status except work schedule was not included in analysis of night shifts.

e P<0.05.

## Discussion

We examined the relation between the change in workers’ symptoms of work stress, night work and nutrient intake in a prospective study design. Our findings suggest that perceived stress, reduced perceived workability, sleepiness and an increase in night shifts were associated with reduced diet quality, and the effect was more often seen among males than females. This study provides new insights into how workers’ increase in experience of stress is associated with diet quality. With the exception of studies on alcohol consumption, we have not found any prospective studies concerning change in stress and nutrient intake.

An increase in stress and decrease in perceived workability was associated with an increase in fat and saturated fat intake among men. Of these associations, change in workability seems to have a stronger influence on fat intake, showing rather consistent 1.3 E% change in fat intake (95% CI 0.57–2.05). Furthermore, increase in sleepiness was associated with a modest increase in saturated fat intake. Similar results have been found in two cross-sectional studies where higher perceived stress was associated with higher proportion of energy from fat in diet (15, 37). Our results accord with earlier cross-sectional studies implicating that stress has a hazardous effect on dietary habits. An increase in saturated fat intake can be detrimental for health because it is an independent risk factor for coronary heart disease (38).

A decrease in vitamin C intake was found among men whose perceived workability was reduced or who were stressed. Change in vitamin C intake may result from lower consumption of fruits and vegetables, which are an important part of healthy diet. We did not find any studies concerning stress and vitamin C intake, but one prospective study showed that stressed workers ate vegetables and fruits less frequently than workers without stress (4). Cross-sectional studies have shown that higher level of stress was associated with lower vegetable and fruit consumption (39, 40). Our results attested the former results and additionally showed that increase in level of stress has an inverse association with vegetable and fruit consumption.

Among women, change in work stress symptoms influenced diet less than in men. Only elevated alcohol intake was associated with an increase in fatigue, but the direction of the association can be speculated as increase in alcohol consumption may worsen sleep quality (41). Also the CI (1.3–13.7) was rather wide, which weakens the reliability of the finding. Both high- and no-alcohol intake were associated with lower work ability in a cross-sectional meta-analysis, with no interactions found in the prospective study design (42). Among women, an increase in stress was associated with an increase in sucrose intake, but this association attenuated after controlling for confounders.

We found that the poorer the quality of men’s diet, the greater the increase in sleepiness. Studies on sleep and diet have focused on the quality and quantity of sleep, and studies on sleepiness and diet are much less common. We found only one study concerning sleepiness and diet, and it showed that day-time sleepiness was associated with abdominal obesity independent of diet and physical activity (43). To our knowledge, only a few studies have investigated associations with changes in sleep patterns and diet. Among men, insomnia symptoms predicted higher energy intake and lower vegetable intake after a few years (44). One longitudinal study explored if changes in diet affect duration of sleep or insomnia symptoms. The authors found that unfavorable changes in diet, evaluated with diet score, increased the possibility of having insomnia symptoms (45). Studies have shown that sleep pattern affects dietary habits, but newer research has suggested the relationship to be two-way. In a study by St Onge et al (46), low fiber and high saturated fat and sugar intake predicted lighter, less restorative sleep with more arousals.

An increase in one night shift resulted in 0.17 E% (95% CI 0.04–0.29) higher saturated fat intake among women but not men. The magnitude of the effect can be considered modest but can nevertheless add to the negative health effect of night work. We were not able to find any studies on the consequences of change in number of night shifts for nutrient intake. However, Vimalananda et al (27) found that a longer history of rotating night shift work reduced diet quality among women, but Pan et al (47) did not find any associations between diet and years worked in rotating night shifts. In these two studies, dietary information was limited to only some foods and nutrients, and it is possible that using a measure of overall diet results in somewhat different findings on the association between night work and diet. In an experimental study, a simulated night shift increased the preference for high fat foods the next morning (48). One study followed novice nurses over two years from the beginning of the first full-time rotating night shift work, and their emotional and uncontrolled eating increased and only modest adaptation was seen (49). Thus, it is possible that an increase in night shifts may increase workers’ strain, which may in turn affect their dietary habits.

We showed that an increase in work stress symptoms has a deteriorating influence on workers’ dietary habits. A possible mechanism behind the relation between stress and quality of diet could be increased cortisol secretion, which stimulates higher levels of energy intake and snacking when confronted by stress (50, 51). Moreover, cortisol can directly stimulate increase in food consumption (52). Acute stress increases cortisol and also appetite-inducing hormones (53) that may induce food cravings (54). In the present study, a sex difference in response to work stress was seen. A recent study showed that responses to acute stress situation were different between sexes as men mounted a greater cortisol response than women (55).

Working environment can have a large impact on workers’ psychosocial health (56). Job-strain-related sleepiness and recovery from work can be reduced with shift arrangements that (i) enable sufficient weekly rest, (ii) avoid short recovery times between shifts or (iii) ensure decent weekly working time (57, 58). This in turn may have an effect on the workers’ diet and long term health.

The present study has several strengths but also limitations. It was a prospective study with over two years follow-up and thus offered a possibility to examine the changes in workers’ stress symptoms, night shifts and nutrient intake. Very few studies have earlier reported this kind of information. It would have been interesting to study association of changes in different types of shift work arrangements with nutrient intake, but – because only very few workers’ changed their work schedule during follow-up – this was not possible. Another option for the analyses could have been dividing the participants in four stress groups according to changes between baseline and follow-up, but there might have been low power to detect differences between the groups in this study population. Therefore, we concluded that the chosen analyses method was the most suitable to study this population. A strength of this study was that we used validated questionnaires for estimating sleepiness (the ESS) and nutrient intake. In addition, the question on stress has been validated and indicates well symptoms of worker stress (34). Moreover, we were able to adjust the results for several covariates. It is possible that covariates changed during follow-up, but, for example, only very few changes were found in occupations or working times. The studied work related factors (stress, work ability, ESS, night work) were self-reported and therefore it is possible that participants have over- or underestimated their symptoms. We cannot exclude potential effects of unmeasured factors, such as sleep apnea, on our results. Participants’ experienced strains might be due to situations other than work in their life. However, participants’ perceived stress and feeling that they were drained from work correlated highly (r=0.65, data not shown). A majority of the participants had increased risk for T2D, and therefore the results may not be generalizable to workers without risk factors for T2D. It is possible that the change in work stress symptoms had a greater impact on the subjects with less healthy lifestyle habits including, those with increased risk for T2D as in the present study. Finally, with our study design we were unable to prove causality, only associations between changes in work stress symptoms, night work and nutrient intake.

In conclusion, increase in night work or work stress symptoms as measured by perceived work stress, workability, fatigue, and sleepiness were associated with changes towards poorer dietary habits among workers. The effect was more often seen among men than women. A healthy diet may lower the risk for chronic diseases and, therefore, occupational healthcare should acknowledge the impact of work stress symptoms on workers’ dietary habits and offer lifestyle interventions for those in need.

## Supplementary material

Supplementary material
