# Supplementary material for: The association of work stress and night work with nutrient intake – a prospective cohort study
Source: Scand J Work Environ Health. 2020 Sep 1;46(5):533–41. doi: 10.5271/sjweh.3899 (PMC7737803; doi:10.5271/sjweh.3899)
Supplement: Supplementary material [file SJWEH-46-533-S001.pdf]

The association of work stress and night work with nutrient intake – a prospective cohort study[1]

by Katri Hemiö, MSc,[2] Jaana Lindström, PhD, Markku Peltonen, PhD, Mikko Härmä, MD, PhD, Katriina Viitasalo, PhD, Sampsa Puttonen, PhD

1. *Supplementary material*
2. *Correspondence to: Katri Hemiö, Department of Public Health Solutions, Finnish Institute for Health and Welfare, P.O. Box 30, FI-00271 Helsinki, Finland. [E-mail: katri.hemio@thl.fi]*

Table S1: Nutrient intake at baseline and nutrient intake change during follow-up, by sex and participation in interventions.

		All (N=366)			All: Men (N=211)			All: Women (N=155)			All: Participants (N=262)			All: Non-participants (N=104)				
		Mean	SD	p-value	Mean	SD	p-value[a]	Mean	SD	p-value[a]	Mean	SD	p-value[a]	Mean	SD	p-value[a]	p-value[b]	95% CI[b]
Sucrose, E%	BL	8.7	2.2	**0.002**	8.4	2.0	**0.03**	9.1	2.4	**0.02**	8.7	2.2	**<0.001**	8.7	2.2	0.52	0.18	-0.12 – 0.65
	change	-0.3	2.0		-0.3	2.0		-0.4	2.1		-0.4	2.0		-0.1	2.1			
Fat, E%	BL	33.0	3.3	0.13	32.7	3.4	0.14	33.3	3.2	0.55	33.0	3.3	0.07	33.0	3.4	0.98	0.25	-0.25 – 0.96
	change	-0.3	3.1		-0.3	3.1		-0.2	3.2		-0.3	3.0		0.0	3.3			
Saturated fat, E%	BL	12.3	1.9	**0.005**	12.1	2.0	**0.002**	12.5	1.9	0.41	12.3	2.0	**0.004**	12.3	1.8	0.47	0.28	-0.14 – 0.49
	change	-2.5	1.7		-0.4	1.6		-0.1	1.7		-0.3	1.7		-0.1	1.6			
Alkohol, g per week	BL	71.4	80.8	**0.004**	91.4	93.3	**0.03**	44.1	47.8	**0.02**	75.1	86.4	**0.01**	62.0	63.5	0.18	0.24	-22.0 – 5.43
	change	+9.6	63.6		+11.9	78.2		+6.4	35.0		+11.1	70.1		+5.7	42.9			
Fibre, g	BL	20.0	4.9	0.25	21.6	5.3	0.78	17.8	3.3	0.07	19.8	4.8	0.90	20.4	5.2	**0.005**	0.12	-1.45 – 0.18
	change	-0.3	4.2		-0.1	4.8		-0.5	3.2		0.0	4.4		-1.0	3.4			
Vitamin D, µg	BL	6.3	2.1	**0.003**	7.1	2.1	0.07	5.3	1.5	**0.01**	6.4	2.2	**0.005**	6.1	1.6	0.30	0.16	-0.61 – 0.10
	change	+0.3	1.8		+0.2	1.9		+0.3	1.6		+0.3	1.9		+0.2	1.5			
Vitamin C, mg	BL	90.0	32.6	**0.002**	84.9	31.7	0.06	96.8	32.7	**0.005**	91.1	34.5	**<0.001**	87.1	27.3	0.74	0.24	-2.13 – 8.38
	change	-4.7	28.1		-4.0	31.0		-5.6	24.4		-6.2	30.0		-0.8	24.7			
Iron, mg	BL	12.4	1.9	0.12	13.5	1.6	0.24	11.0	1.0	0.28	12.4	1.9	**0.02**	12.5	1.7	0.19	0.07	-0.59 – 0.02
	change	+0.1	1.5		+0.1	1.7		+0.1	1.3		+0.2	1.6		-0.1	1.1			

[a] Statistical significance for change in nutrients intake within the group

[b] Statistical significance and 95% confidence interval for change in nutrients intake between the lifestyle intervention participants and non-participants, analyses adjusted for baseline nutrient intake and sex
